# Does diet influence aging? Evidence from animal studies

**DOI:** 10.1111/joim.13530

**Published:** 2022-06-24

**Authors:** David G. Le Couteur, David Raubenheimer, Samantha Solon-Biet, Rafael de Cabo, Stephen J. Simpson

**Affiliations:** 1 Charles Perkins Centre, The University of Sydney, Sydney, Australia; 2 ANZAC Research Institute, The Concord Hospital, Concord, Australia; 3 School of Life and Environmental Sciences, The University of Sydney, Sydney, Australia; 4 Translational, Gerontology Branch, Intramural Research Program, National Institute on Aging (NIH), Baltimore, Maryland, USA

**Keywords:** aging, caloric restriction, fasting, nutritional geometry, obesity

## Abstract

Nutrition profoundly influences the risk for many age-related diseases. Whether nutrition influences human aging biology directly is less clear. Studies in different animal species indicate that reducing food intake (“caloric restriction” [CR]) can increase lifespan and delay the onset of diseases and the biological hallmarks of aging. Obesity has been described as “accelerated aging” and therefore the lifespan and health benefits generated by CR in both aging and obesity may occur via similar mechanisms. Beyond calorie intake, studies based on nutritional geometry have shown that protein intake and the interaction between dietary protein and carbohydrates influence age-related health and lifespan. Studies where animals are calorically restricted by providing free access to diluted diets have had less impact on lifespan than those studies where animals are given a reduced aliquot of food each day and are fasting between meals. This has drawn attention to the role of fasting in health and aging, and exploration of the health effects of various fasting regimes. Although definitive human clinical trials of nutrition and aging would need to be unfeasibly long and unrealistically controlled, there is good evidence from animal experiments that some nutritional interventions based on CR, manipulating dietary macronutrients, and fasting can influence aging biology and lifespan.

## Introduction

The world’s population is aging rapidly, leading to accelerating numbers of people with age-related diseases and impaired physical and cognitive function. “Healthy aging” has been promoted as a way to postpone and minimize the effects of aging and is based on the prediction that a healthy lifestyle, coupled with an accessible health care system and age-friendly environments, will increase the period of healthy life and reduce the period of poor health in old age [1, 2].

Exercise and good nutrition are principal components of healthy aging lifestyles. The links between diet and diseases in human populations are well established, especially for diseases secondary to nutritional deficiencies and obesity [3]. The association between diet and human aging is less clear. Here the evidence relies mostly on animal data, because well-controlled human clinical trials of the effects of nutritional interventions on aging and lifespan are not feasible. The keystone finding in animal studies is that “caloric restriction” (CR), which involves reducing the amount of food (dietary calories) provided to animals, increases their lifespan compared to those with free access to food (ad libitum fed) [4–7]. CR has been the foundation for recent research that has attempted to identify other nutritional interventions that can influence the aging process, and might be more acceptable to humans than CR.

From the experimental perspective, it is challenging to characterize the relationship between nutrition and aging, and to prove that diet influences aging at the mechanistic level. The reasons for this are related to how aging is defined and measured, and how dietary interventions are designed and interpreted.

### The definition and measurement of aging

Most definitions of aging propose that aging is a progressive process associated with decline in structure and function, impaired maintenance and repair systems, reduced reproductive capacity, and increased susceptibility to diseases and death [8]. It has been difficult to use these broad concepts to create precise outcomes that can be operationalized in experimental studies.

Animal studies over the last century have generally relied solely on lifespan to define aging [9, 10]. Death is usually the result of disease and trauma, and although aging is the most inescapable risk factor for death, it is rarely diagnosed as the cause of death. However, aging is often considered to be a risk factor for many diseases, particularly those chronic noncommunicable diseases that increase exponentially with age such as dementia and ischemic heart disease [11]. Alternatively, aging and aging-related diseases might simply be early and later stages on a continuum of pathological and phenotypic characteristics [12, 13].

Many studies now report both lifespan and disease. In studies of mice, diseases commonly include tumors found at post mortem and cardiometabolic diseases evaluated in vivo. Most dietary interventions that lead to increased lifespan nearly always delay the onset and burden of disease as well [14–16]. When a diet increases lifespan and reduces aging-related diseases, it is probably reasonable to conclude that it is acting on aging, because aging is a risk factor for disease.

Lifespan is not a feasible aging outcome for human studies of dietary interventions. A longterm, double-blind, randomized clinical trial in humans of a nutritional intervention with lifespan as an outcome, is unlikely ever to be attempted or successfully undertaken. If randomized clinical trials are put forward as the only way to prove the efficacy of any intervention [17] then the effects of diet on aging and lifespan are never likely to be proven in humans. Determining age-specific mortality can provide additional insights from observational studies [18]. Even so, evidence to support the use of diets to delay aging in humans is more likely to be provided by animal trials in combination with observational studies and short-term clinical trials measuring surrogate aging biomarkers in humans.

There has been an intensive search for aging biomarkers that could be surrogate outcomes for aging and lifespan [19]. The ideal aging biomarker should be directly linked to some aspect of aging biology. The biological processes of aging have been cataloged as the nine “Hallmarks of Aging” and these are potential aging biomarkers. The Hallmarks include changes in DNA that are the likely initiators of aging (genomic instability, telomere attrition, epigenetic changes/DNA methylation) and lead to changes at the subcellular (loss of proteostasis, mitochondrial dysfunction, deregulated nutrient sensing) and at the cellular level (cellular senescence, stem cell exhaustion, altered intracellular communication) [20]. Aging biomarkers most often used in dietary studies have been DNA methylation, which increases with age [21, 22] or telomere length, which decreases with age [23]. Both are referred to as aging clocks.

Another approach to discovering aging biomarkers is to measure many parameters in a cohort and use statistical methods to identify those, either individually or in composite sets, that perform best as biomarkers of aging [24]. However, there are no gold standards for defining or validating aging biomarkers. One standard that has been suggested is that an aging biomarker should be able to predict some functional capability at some later stage better than chronological age [25]. Even so, candidate biomarkers are often validated directly or indirectly against chronological age, lifespan, or remaining life expectancy. Many aging biomarkers are also risk factors for, or measures of severity of, diseases (e.g., NT-proBNP, HbA1c, insulin, cystatin-C) and these could be thought of as biomarkers of disease rather than aging [26]. Clinical syndromes such as frailty, sarcopenia, health span, and multimorbidity are also being explored as aging biomarkers [26]. The advantage of these conditions is that they are valid standalone outcomes, as well as biomarkers of aging.

To overcome these challenges in measuring aging, there has been a move towards data-driven approaches to interpret the relationship between nutrition and aging. Such approaches may involve thousands of mice and collection of in vivo and in vitro data and samples at multiple time points over the lifespan. Interpretation is data driven and can depend on machine learning and complex modeling [27–34].

When multiple biomarkers have been measured, another approach is to estimate “physiological dysregulation.” This is calculated from the Mahalanobis distance (D_M_), which is a single value that incorporates the differences between each biomarker and a statistical norm, converting a multidimensional biomarker space to a single dimension [35, 36]. D_M_ increases with age in a similar way across primate species, including humans [37], and has recently been used to evaluate the effects of diet on aging in humans. In a population of older people, the relationship between D_M_ for several measures of healthy aging, macronutrients, and micronutrients was usually nonlinear and interactive, and no “good” or “bad” macronutrient for aging could be identified across all domains [38].

### Design and interpretation of diet studies and aging

Standard laboratory diets and conditions are very different from those that shaped the evolution of most model laboratory animals. In the wild, animals select from a range of foods to meet dietary targets for the intake of energy, macronutrients, and micronutrients. These dietary behaviors have evolved to optimize Darwinian fitness in an environment where animals are active as they forage for food, the temperature is not stable, and the availability and types of food vary [39, 40]. In the wild, mice can have many small meals eaten at night and foraged from 20–30 different food sites [41]. In animal laboratory studies, there is ad libitum access to a single type of diet, stable temperature, and limited physical activity.

In rodent studies, standard chow was developed and modified in 1993 by the American Institute of Nutrition to standardize diets (AIN-93G, AIN-93M). These are reconstructed diets with high sucrose content for ease of pelleting, and they lack soluble fiber. Unlimited access to these diets in rodents maintained in laboratory conditions can cause obesity, fatty liver, and metabolic dysfunction, and increase oxidative stress and cancers [42–45]. Dietary experiments often compare animals fed a control diet such AIN-93 to those on an experimental diet, which has been generated by the addition or removal of one particular nutrient to the control diet. This methodology is known as the one-variable-at-a-time (OVAT) approach and has been useful in establishing the clinical features of vitamin deficiencies, but has been much less successful with complex conditions and chronic noncommunicable diseases [46]. An exemplar is CR, where the control group has unlimited access to food, while the intervention group receives less food. The OVAT interpretation would be that any increase in lifespan is attributed only to the reduction in dietary calories compared to the control diet.

Diet is a complex mixture and the OVAT approach doesn’t account for interactions between dietary constituents, nor nonlinear relationships between diet and phenotype. For example, some studies conclude that a low protein diet increases lifespan. However, when all three macronutrients are analyzed, diets that are low in protein and high in carbohydrates increase lifespan, while diets that are low in protein and high in fat are linked with poorer health outcomes [16].

### Nutritional geometry

Nutritional geometry is an experimental methodology and interpretive platform that can resolve many of these issues [39, 46–49]. Nutritional geometry studies usually measure the outcomes from a large number of diets (typically 20–30) that systematically explore dietary dimensions of interest, such as caloric density and macronutrient ratios. Analysis is control-agnostic, because there is no single “control” diet. Instead, the outcomes are measured over a range of dietary constituents. The outcome is presented as a heatmap or surface spread across the entire topology of dietary compositions and nutrient intakes.

**Fig. 1:**
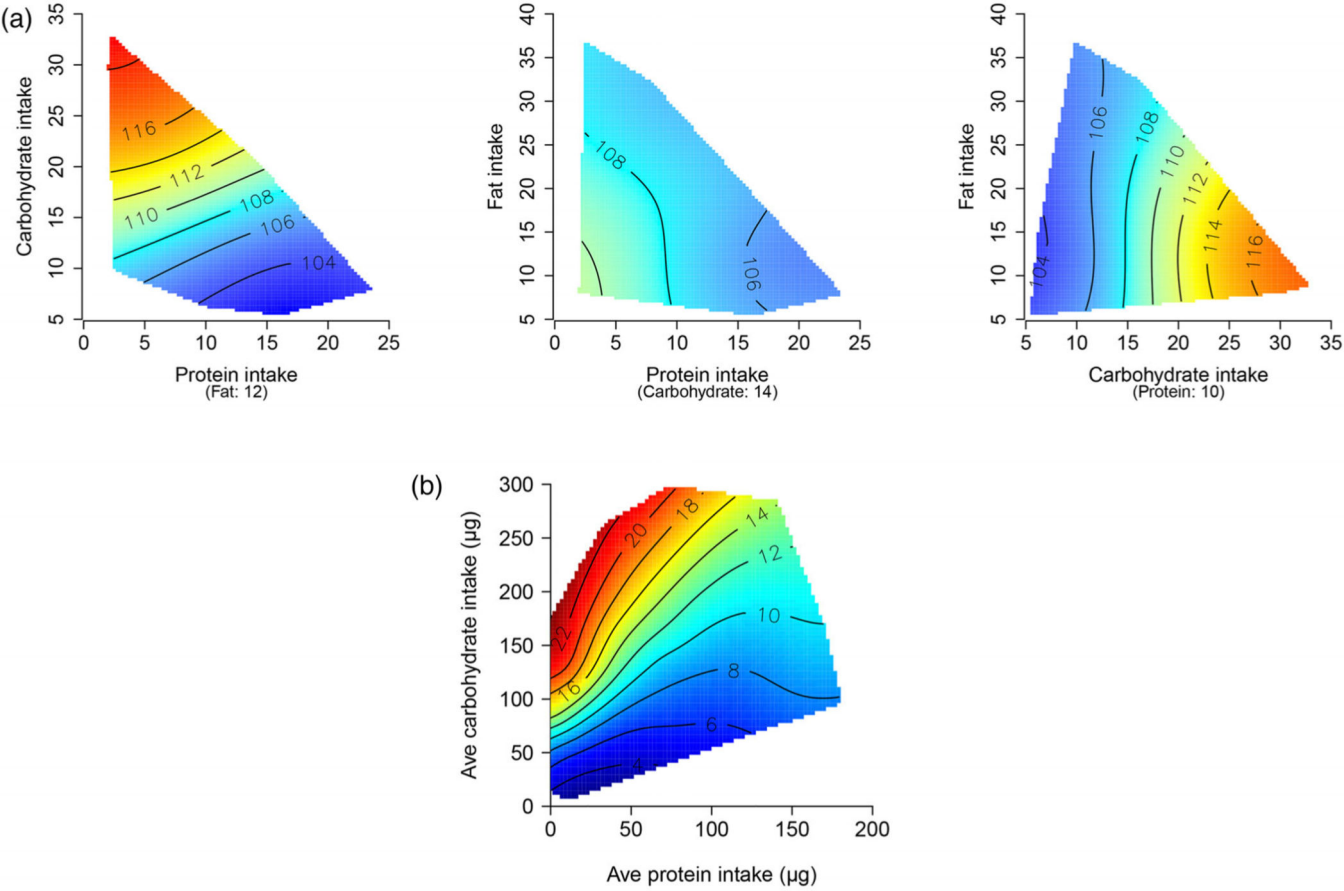
Macronutrients and lifespan in (a) mice and (b) Drosophila. In these studies, Drosophila and mice had ad libitum access to one of 28 and 25 diets, respectively, varying in macronutrients and energy content. Lifespan data are presented as heatmaps ranging from blue (lowest values) to red (highest values). Drosophila diet has two macronutrients, protein and carbohydrates; therefore, one heatmap is shown with the axes representing the intake of carbohydrate or protein. In mice, there are three macronutrients—protein, carbohydrates, and fat; therefore, three heatmaps are presented, each having two macronutrients for the x and the y axes, at the median value of the other macronutrient. In both Drosophila and mice, the highest lifespan (red area of heatmaps) was the longest with diets that were higher in carbohydrates, lower in protein, and where the energy intake was higher (adapted from Lee et al. [155] and Solon-Biet et al. [16]).

**Fig. 2:**
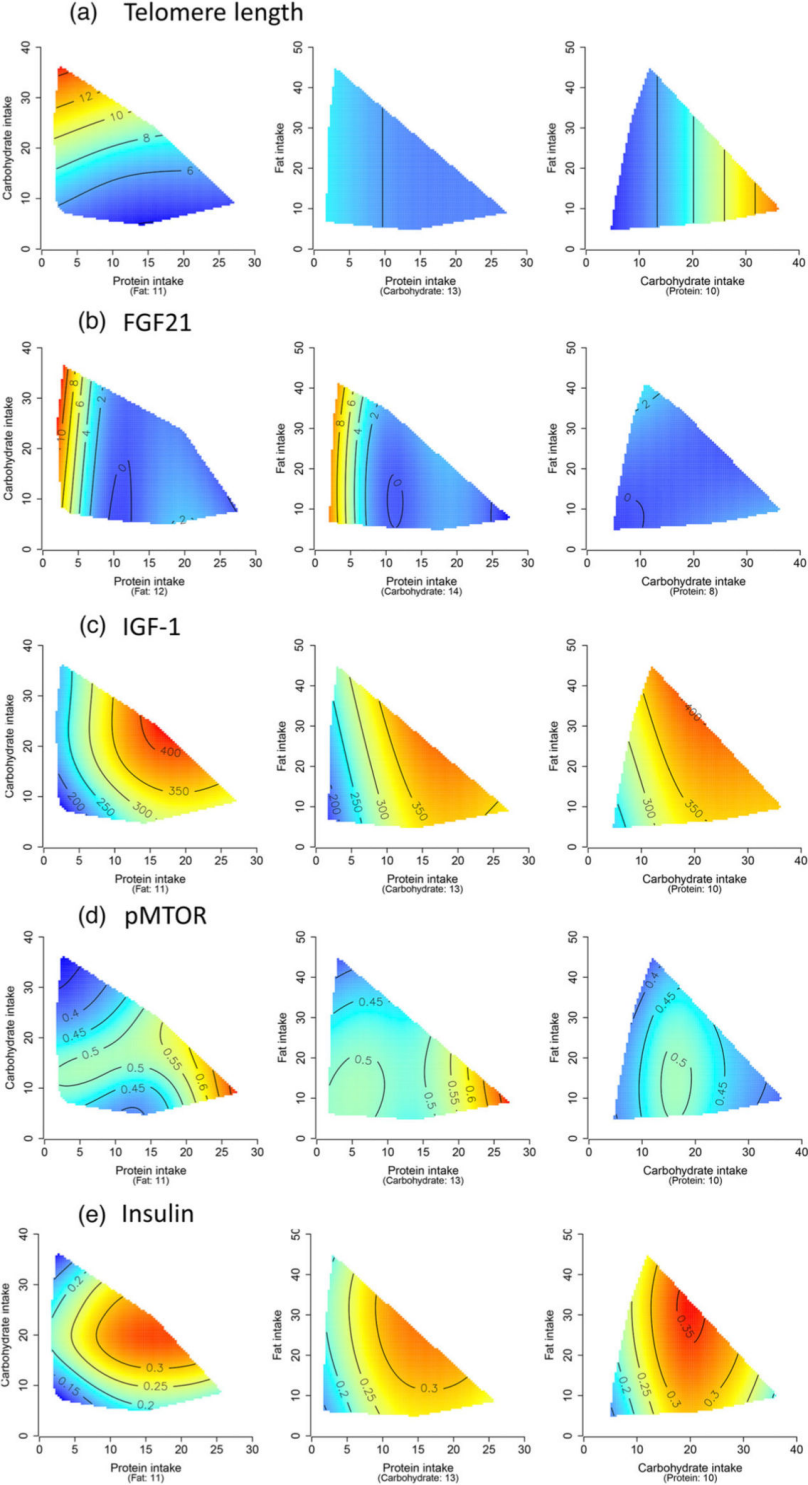
Macronutrients and (a) telomere length in the liver, (b) FGF21 levels, (c) IGF-1 levels, (d) phosphorylated mechanistic target of rapamycin (MTOR) in the liver, and (e) insulin levels. Data are presented as heatmaps ranging from blue (lowest values) to red (highest values). Three heatmaps are presented, each having two macronutrients for the x and the y axes, at the median value of the other macronutrient.

In mouse studies of macronutrients, the heatmap is a three-dimensional surface with the axes consisting of the intakes of protein, carbohydrates, and fat (Figs 1 and 2). The topology (patterns of color) of the response surfaces, supported by appropriate statistical models such as generalized additive models, indicate which macronutrients and in which amounts and combinations impact outcomes. Quantifying the shape of the response surface provides important information about the main and interactive effects of nutrients, which is unavailable using OVAT approaches. These surfaces can be mapped either as a function of the intakes of dietary constituents (intake based), or their concentration in the diet (diet based) [50]. Intake-based analyses allow account to be taken of any effects of compensatory feeding [48, 51]. For example, a low-protein diet may not lead to a reduction in protein intake because the animal will eat more food to reach its protein target, at the cost of overconsumption of fat and carbohydrates. This is called “protein leverage” [52].

### CR and aging

CR has been a pillar of aging research for nearly a century. There are many reports that reducing food intake lengthens lifespan. It may also postpone the onset of aging-related diseases [14], protect against carcinogenesis [53], and delay many of the biological changes associated with aging [54]. Up until the turn of the century, CR was utilized mostly in attempts to identify and characterize mechanisms for aging [10]. More recently, CR has been used to discover pathways that can be targeted by pharmacotherapies that might have beneficial effects on aging by replicating CR [55, 56].

The 1935 publication by McCay et al. is often considered to be the first seminal work linking CR with lifespan. McCay believed that retarding growth by limiting calorie intake would influence lifespan. In one group of white rats, food intake was reduced so that their body weight at 1 year was about 100 g compared to about 300 g for the other group that had free access to food. The average lifespan was increased from about 500 days to over 800 days in the calorically restricted male rats [57].

Since 1935, there have been thousands of publications on CR in a wide range of taxa, and recent meta-analyses of these studies. In 2012, Nakagawa et al. [58] published a meta-analysis of 145 studies in 36 species. CR reduced the risk of death by 60% and there was a U-shaped relationship between the degree of CR and risk of death, which was minimized when calorie intake was reduced by about 50%. The effects were 20% less in males than females, and greater in “model” laboratory animals (*Saccharomyces cerevisiae*, *Caenorhabditis elegans*, *Drosophila melanogaster*, *Mus musculus*, and *Rattus norvegicus*). They also reported the effect of protein restriction on risk of death was greater than that seen with CR. Risk of death was lowest when protein intake was reduced by about 30%.

Swindell [9], also in 2012, meta-analyzed data from 53 studies in rats and 72 studies in mice that had been published since 1934. He concluded that CR increased median lifespan by 30.4% and maximum lifespan by 32.3% in rats. In mice, CR increased median lifespan by 14.6% and maximum lifespan by 17.8%. He noted significant effects of mouse strain on the effects of CR. The most consistent effects were in C57Bl6 mice. Inconsistent or even negative effects were seen in C3H and DBA strains and some of the recombinant inbred ILSXISS strains. Although the conclusion was that CR increased lifespan, caveats were that the degree of lifespan extension was less than previously reported, was dependent on the strain, and possibly limited to rodents maintained in laboratories.

Speakman and colleagues subsequently provided a detailed characterization of the effects of graded levels of short-term CR in C57Bl6 mice [31–34, 59–69]. There were four groups of calorically restricted mice that received 17%, 24%, 35%, and 44% less calories compared to ad libitum fed controls (10%, 20%, 30%, and 40% compared to their baseline intake). The diets were administered for 12 weeks. These studies confirmed that CR is associated with reduction in fat and body temperature, and changes in some hormones—reduced levels of leptin, TNF*α*, IGF-1, and insulin. CR was associated with changes in metabolome and transcriptome in blood, liver, hypothalamus, epididymal fat, and cerebellum. These changes were mostly consistent with an effect on aging and hunger pathways. The effects sizes of these outcomes tended to increase with the degree of CR.

Amongst the most influential recent studies on CR are two rhesus monkey studies [14, 70, 71]. These studies were commenced in the 1980s at the University of Wisconsin (UW) (*n* = 76) and the National Institute on Aging (NIA) (*n* = 121). The NIA study used a naturally sourced diet that was lower in fat and sucrose and higher in protein and fiber than the semipurified diet used in the UW study. The NIA study supplemented both control and CR groups with vitamins while the Wisconsin study only supplemented the CR group. CR significantly increased lifespan in the UW by approximately 5%, but there was no significant change in the NIA study. CR reduced the rate of onset of age-related conditions by about twofold and improved glucose insulin metabolism in both UW and NIA monkeys. Because of similarities in the aging of rhesus monkeys and humans, the authors of both studies were confident that the health benefits of CR, if not lifespan extension, would be translated into humans [70].

There has been one randomized clinical trial, albeit relatively short term, of CR and aging in humans. The CALERIE study was designed to evaluate the effect of 25% reduction in calorie intake over 24 months in nonobese humans. The outcomes were chosen to reflect aging. There were some findings in calorically restricted humans that were similar to results from animal studies of CR. These included reduced body temperature, insulin levels, and DNA oxidation [72], increased mitochondrial biogenesis in muscle [73], and reduced oxidative stress determined from urinary isoprostanes [74]. CR led to a reduction in cardiovascular risk factors [75] but also adverse outcomes such as reduced bone mineral density [76].

A DNA methylation biomarker of aging developed in the Dunedin study, called DunedinPoAm, was evaluated in the CALERIE participants after 12 months on their diets. The rate of change of this biomarker in the control group was 0.71 years of biological change per year of follow-up. In the CR group, the rate of change was 0.11 years of biological change per year of follow-up and this was significantly less than the control group [77]. This post hoc analysis suggests that CR in humans directly influences at least one Hallmark of biological aging—DNA methylation. It also indicates that this DNA methylation biomarker could be an effective surrogate outcome for aging in future human trials of diet and aging.

Another recent study in humans revealed the potent effects of CR on the intersection between the immune system and metabolism in humans. CR led to an improvement in thymopoiesis that was correlated with the mobilization of intrathymic ectopic lipid [78].

### The effects of CR on the Hallmarks of Aging in animal models

CR delays or prevents all the Hallmarks of Aging [54]. Below are examples of the positive effects of CR on the Hallmarks of Aging.

#### Genomic instability

Aging is associated with genome instability, which promotes both aging and carcinogenesis [79]. Genetic conditions with defects in DNA repair such as Werner’s syndrome and Hutchinson Guildford Syndrome cause premature aging. DNA damage directly influences the other Hallmarks of Aging, including cellular senescence, mitochondrial function, proteostasis and autophagy, stem cells, and nutrient signaling [79]. CR improves genomic stability and reduces DNA mutations by enhancing DNA repair pathways, including base excision repair, nucleotide excision repair, and nonhomologous endjoining [54, 79–81].

#### Telomere attrition

Telomeres shorten with every cell division and the length of telomeres correlates with the number of cell divisions, hence also age. After a certain number of cell divisions (50–70 in humans), telomeres are sufficiently short such that cell division ceases, and cells become nondividing senescent cells. These cells secrete inflammatory cytokines called senescence-associated secretory phenotype (SASP). There is limited evidence about the effects of CR on telomeres. CR reversed age-related reduction of telomeres in the lens epithelial cells of rats [82] and blood leukocytes in mice [54, 83].

#### Epigenetic changes

Aging influences DNA methylation and histone remodeling, the main epigenetic regulators of gene expression [84]. Epigenetic clocks refer to patterns of DNA methylation at CpG sites that correlate with age. They include the Horvath clock, PhenoAge, DunedinPoAm, and GrimAge [77, 85]. The transcriptome also reflects epigenetic regulation. CR prevents age-related changes in DNA methylation in the blood, liver, kidney, and brain of mice, while mice on CR have younger epigenetic clock ages compared with controls [54, 84]. Histone remodeling is regulated by the histone deacetylase sirtuin1 (SIRT1) and nicotinamide adenine dinucleotide, which may be master regulators of the response to CR.

#### Loss of proteostasis

Proteostasis refers to the processes that regulate and maintain structurally intact proteins in cells. This involves protein translation and removal of damaged proteins via mechanisms such as the proteosome, autophagy, and unfolded protein responses. Aging is associated with impaired protein translation, autophagy dysfunction, and, as a consequence, intracellular accumulation or aggregation of misfolded proteins. One of the key effects of CR is to increase autophagy; however, the reports about the effect on proteosome are inconsistent [54]. For example, in one pivotal study in mice, CR reduced the accumulation of polyubiquitinylated proteins by increased autophagy but without any impact on the expression of activity of the components of the proteosome [86].

#### Mitochondrial dysfunction

Aging influences many aspects of mitochondrial biology, including the numbers of mitochondria in each cell, biogenesis, mitochondrial ultrastructure, oxidative phosphorylation, ATP synthesis, generation of reactive oxygen species, mitochondrial DNA mutations, fusion/fission, mitophagy, and transport across mitochondrial membranes [87–89]. CR delays the age-related decline in mitochondrial numbers and function [54, 90]. One of the most important proteins upregulated by CR is peroxisome proliferator-activated receptor *γ* coactivator 1*α* (PGC-1*α*), which is a master regulator of mitochondrial biogenesis and function [91]. The reduction ATP synthesis coupled with increased oxidative stress that occur with aging are prevented by CR [54, 89]. Mitochondria are necessary for the beneficial effects of CR [86].

#### Cellular senescence

Senescent cells have stopped dividing indefinitely, for example, as a result of age-related telomere erosion. They accumulate with age and some senescent cells secrete inflammatory cytokines, the SASP [92]. CR in mice reduces the numbers of senescent cells in the liver, intestines, and brain [93] and delays senescence in T cells [94].

#### Stem cell exhaustion

All the Hallmarks of Aging become apparent in aging stem cells, leading to lower numbers of stem cells and more senescent stem cells [95, 96]. Transplantation of mesenchymal stem cells from young donors to older people may provide a solution to frailty and sarcopenia [97]. CR maintains stem cells and reduces senescent cells in the brain [98] and intestine [99] of mice.

#### Intercellular communication

The main example of age-related changes in intercellular communication is inflammation. Aging is associated with low-grade inflammation with activated macrophages and elevated levels of inflammatory cytokines and markers (interleukin 6, tumor necrosis factor alpha, C-reactive protein), which has been termed “inflammaging” [100]. Aging is also associated with impaired innate immune responses secondary in part to thymic atrophy and T-cell depletion, termed “immunosenescence” [101]. CR reduces many components of age-related increase in inflammation [102, 103], including inflammatory pathways in adipose tissue [65] and increased levels of interleukin 6 and tumor necrosis factor *α* [104].

#### Deregulated nutrient sensing

Nutrient sensing pathways are those that detect changes in nutrients such as energy, protein, carbohydrates, and fat, and regulate a vast network of downstream cellular pathways that need to be nutrient responsive. These pathways have been a major focus for CR research, driven by the promise that drugs acting on nutrient sensing pathways may be able to replicate some of the beneficial effects of CR without the need to reduce food intake. Nutrient sensing pathways tend to be similar across taxa and demonstrate age-related changes. Genetic and pharmacological manipulation of some of these pathways have been found to influence the response to CR and lifespan [105–107].

The pathways that have been the main focus for research on aging and CR are the somatotropic axis and the mechanistic target of rapamycin (MTOR) pathway. The somatotropic axis involves the growth hormone (GH), insulin-like growth factor 1 (IGF-1), and insulin. Aging is associated with down regulation of this pathway with insulin resistance and declining levels of GH and IGF-1 [108], and genetic manipulation of proteins in this pathway has extended lifespan in worms, flies, and mice [106, 107]. Mice with knock out of the GH receptor (GHRKO mice) [109] have the greatest extension of lifespan achieved with any intervention to date [109]. Reduction in insulin levels and improved glucose tolerance are amongst the most consistently reported outcomes of CR [29, 110, 111].

The MTOR pathway is activated primarily by amino acids, especially leucine, and one of its major actions is to regulate DNA translation and protein synthesis. MTOR also responds to other growth factors and energy status and regulates many other downstream targets such as autophagy, insulin/glucose metabolism, mitochondria, and the cytoskeleton [106, 107, 112, 113]. Inhibition of MTOR with rapamycin increases lifespan in worms, flies, and mice (by 9% in male mice and 13% in female mice) [112, 114]. CR and rapamycin have both similar and divergent effects in mice. CR leads to a significant reduction in MTOR signaling; however, there are differences between CR and rapamycin in terms of their effects on insulin sensitivity and the expression of cell cycle and sirtuin pathway genes [115], and overall changes in gene expression [116]. Inhibition of these two nutrient sensing pathways increases lifespan.

By contrast, two other nutrient sensing pathways are activated by CR [106, 107, 117]: the adenosine monophosphate-activated protein kinase (AMPK) pathway and sirtuin pathways. The AMPK pathway responds to cellular energetics by sensing the ratio of AMP/ATP while metformin (at a lower dose of 0.1% w/w, but not higher doses), which increases AMPK activity, increases lifespan in mice [118]. Resveratrol, which has as part of its mechanism the activation of SIRT1, increased lifespan in mice on a high-fat diet [119], but not those on a standard diet [120].

If it is accepted that the Hallmarks of Aging are an accurate representation of the biological processes that lead to aging, then the robust finding that CR has an impact on each of the Hallmarks can be considered to be evidence that CR is acting on the aging process.

### Obesity, aging, and CR

It has been proposed that CR does nothing more than prevent obesity-related mortality that occurs in laboratory rodents [121–123]. This has been called the “laboratory glutton” hypothesis. This hypothesis rejects the possibility that CR acts directly on the aging process [123].

Laboratory mice restricted to standard laboratory foods may overeat and become overweight. This is an important issue for all mouse research [123]. However, this observation by itself does not disprove the hypothesis that CR acts on aging, since there is no a priori reason why CR would not act on aging in mice, whether obese or nonobese. The laboratory glutton hypothesis of course does not apply to the effects of CR in animals that are not obese. An informative example is the Ercc1^Δ/−^ mouse, which is a progeroid DNA repair deficient mouse with low food intake and low body weight. In this mouse, CR dramatically increases lifespan [124].

Another argument for the laboratory glutton hypothesis is that there is a negative correlation between body weight and lifespan in obese mice, therefore reducing body weight should increase lifespan [121]. There are mouse studies proving that weight reduction in obese mice increases lifespan [125]. However, weight loss doesn’t always correlate with increased lifespan in CR experiments. For example, in one strain of mice, 40% CR led to greater weight loss than 20% CR, yet lifespan was longer in the mice with 20% CR [86]. Likewise, in one study, weight cycling generated by intermittent fasting increased lifespan without weight loss [125], although a recent meta-analysis did not detect health benefits with weight cycling in rodents [126].

Regardless, the fact that reducing calorie intake leads to increased lifespan is consistent with both an aging mechanism and the laboratory glutton mechanism. The evidence supporting either mechanism is the same—reducing calorie intake increases lifespan.

The reason that CR increases lifespan in both aging and obesity is most likely because of similarities between aging and obesity (Fig. 2). Obesity has been characterized as a type of accelerated aging [127–130] and all the Hallmarks of Aging are found in obesity [131]. A recent study reported that obesity in adolescence is a risk factor for aging in middle age where aging was determined from the pace of aging score, gait speed, brain age, and facial age [132]. Studies of anti-aging drugs, such as resveratrol, have used high-fat diets in mice as a model of aging [119, 133]. On the other hand, aging is associated with accumulation of body fat and impaired glucose and insulin tolerance, which are typical features of obesity. Wolf, in a strong critique of CR, drew the same conclusion: “alleviation of obesity … tells us something about the fundamental mechanisms of aging” [121].

However, the relationships between caloric intake, obesity, and aging can be dissociated depending on the macronutrient composition of the diet. In a study on the effects of macronutrients and dietary energy on lifespan in mice, it was mice on the diets that were lower in protein and higher in carbohydrates that had the longest lifespans. Yet mice on these diets also had higher body weights as a result of increased energy driven by protein leverage [134].

**Fig. 3:**
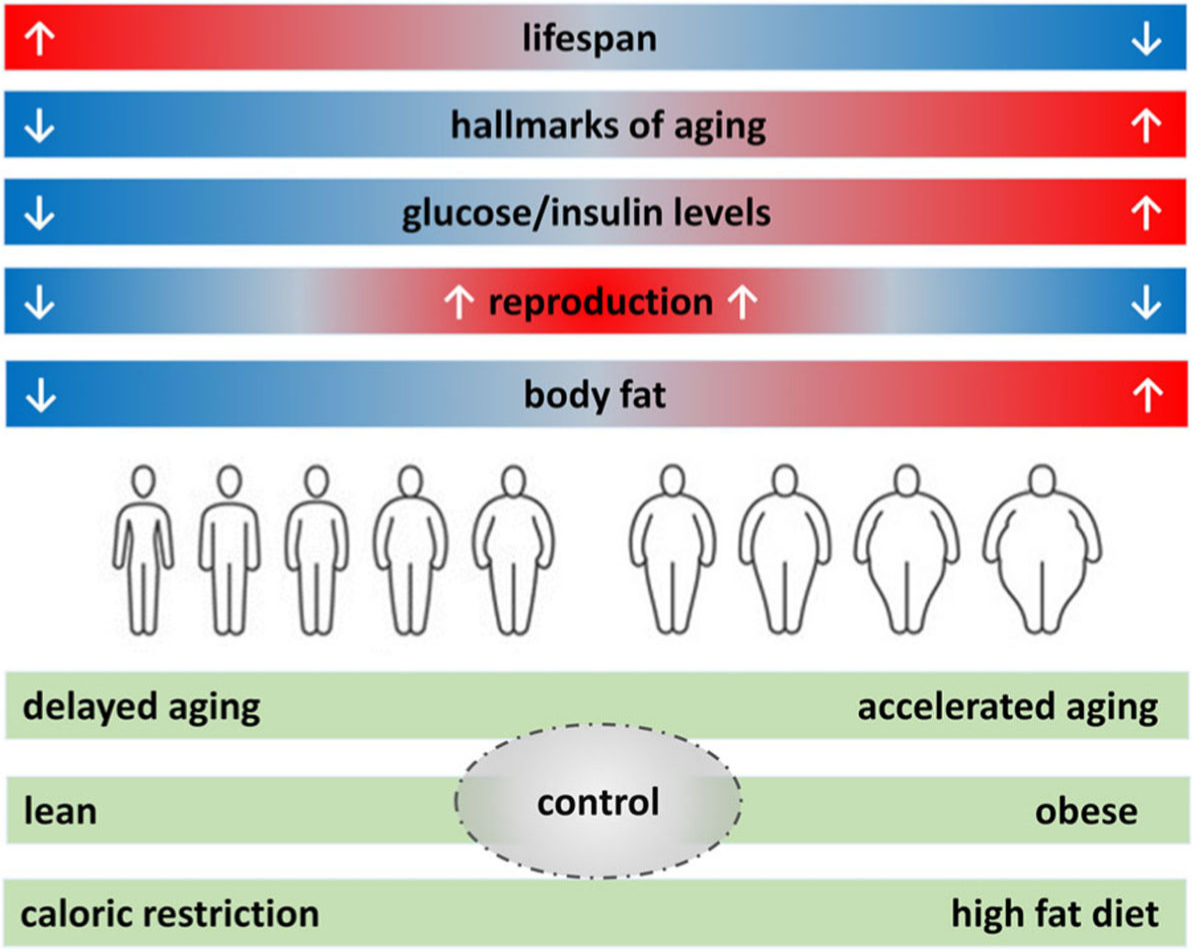
Aging, obesity, and calorie intake. Obesity can be considered to be accelerated aging with reduced lifespan, increased body fat, abnormal glucose/insulin metabolism, and increased evidence of the Hallmarks of Aging. Caloric restriction (and fasting) causes delayed aging and has the opposite effect on these parameters. Reproductive function is reduced by both caloric restriction and obesity; therefore, its optimum diet and body weight must be at some point in between (U-shaped relationship). It should be noted that (1) these are biomedical endpoints pertaining to model systems and (2) the relationship between obesity and aging can also be influenced by the macronutrient composition of the diets.

The debate about whether CR in rodents prevents obesity or delays aging needs to be reframed. The key issue is that overall, reducing caloric intake most likely improves health and increases lifespan in both aging and obesity because of the similarities between them, rather than differences (Fig. 3).

Disagreement about the mechanisms of CR can be viewed as a consequence of methodology where an intervention diet is compared to a control diet. The conclusions drawn by this approach are influenced by assumptions about what defines a control diet and a control level of caloric intake, and where the cut off is between obese and nonobese mice.

The assumptions around the choice of the control group and control diet are complex. As described above, laboratory mice are fatter and inactive compared to wild mice, and they develop a range of obesity-related diseases. Therefore, it has been concluded that standard laboratory rodents are not appropriate controls for studies of CR and aging [45, 121]. Alternatively, it could be argued that an overweight sedentary mouse is an ideal control because it reflects prevalent human characteristics in modern society. This might seem to be flippant, but it underscores the unsolved problem of defining a control animal and control group in nutritional research.

From the evolutionary perspective, the closest to a control diet would be one that meets all the nutrient targets without any need to compromise, or to substantially overconsume or underconsume any of the nutrients. However, these intake targets are likely to change as an animal goes through different phases of life or is challenged by infections and disease [135, 136]. Identification of intake targets requires sophisticated modelling and has not been used to justify the choice of control diets in CR experiments [47].

The nutritional geometry approach avoids this issue because it interprets all the data across all the diets and phenotypes and avoids the constraints caused by labelling as “controls” one group of mice on one type of diet. Nutritional geometry emphasizes the spectrum of changes caused by CR across diets and phenotypes. Whether the control groups are obese or not is irrelevant because they are just another datapoint across a range of body weights.

### CR and fasting

Recent studies on the health outcomes of fasting and CR have shown that CR is secondary to more than just a reduction in calorie intake. The traditional models of CR that increase lifespan involve giving mice a single aliquot of food each day, which they rapidly consume. These mice have periods of fasting between meals. An alternate approach to CR is where mice have free access to food that has been diluted with nondigestible fiber. Mice on these diets are calorically restricted but are not fasting and do not have an increased lifespan [16, 137]. Consequently, there has been growing interest in fasting as an approach for increasing lifespan, particularly since it might be better tolerated in humans than CR [137–139].

Intermittent fasting improves insulin and blood glucose to the same extent as standard CR despite maintaining the same calorie intake and body weight as mice with free access to food [140]. A recent study that compared multiple feeding regimes concluded that many of the effects of CR, including lifespan extension and improved insulin glucose metabolism, are mediated by fasting rather than a reduction in calories [137]. Weight cycling in mice caused by intermittent fasting led to an increased lifespan despite no loss of body weight [125]. It should be noted that fasting is also effective in obesity and obesity-related conditions [141–143].

### Macronutrients and the geometric framework

A key question is whether CR is mediated by reduction in calories, or by reduction in one or more of the macronutrients (protein, fat, or carbohydrates), acting singly or interactively. In CR experiments, it isn’t possible to differentiate the effects of reducing calories from reducing any of the macronutrients because they are all reduced by the same proportion.

Dietary protein was the main focus of early studies because it was thought that CR increased lifespan by delaying growth, and it was known that protein was important for growth. Most studies [144–149] but not all [150–152] found that reducing the protein content in the diet led to a longer lifespan in ad libitum fed animals. However, CR had a greater effect on lifespan than ad libitum fed low protein diets, even when both groups consumed the same amounts of protein [153]. Conversely, the increase in lifespan with CR was not diminished by a high protein diet [154]. These results indicated that although low protein diets increase lifespan, the main mechanism for CR is a reduction in calorie intake, not protein intake.

Nutritional geometry provided a different approach to resolve this issue [39]. This methodology allows the effects of dietary energy and macronutrients and their interactions to be interpreted in the same set of experiments. It doesn’t require a control value for energy intake in order to assess the effects of CR, which as discussed above, can be problematic when the control animals overeat and become obese, as might be the case for laboratory mice.

The first so-called “geometric framework” study of lifespan used the nutritional geometry approach in *Drosophila* that had ad libitum access to one of 28 diets varying in the amount of protein, carbohydrates, and energy. The longest lifespans were in flies consuming diets low in protein and high in carbohydrates (Fig. 1). Lifespan was longest when the ratio of the dietary intake of protein–carbohydrate was 1:16, while CR per se had no beneficial effect on lifespan [155]. The same result was found in subsequent studies by others in *Drosophila* [156], Queensland fruit flies [157, 158], crickets [159], and ants [160].

A geometric framework study was then undertaken in mice (Fig. 1). Mice had ad libitum access to one of 25 diets containing different amounts of protein, carbohydrates, fat, and energy. CR was achieved by diluting the food with nondigestible fiber and the mice eating these diets had about 30% less energy intake than those eating nondiluted diets. Similar to the insect studies, the longest lifespans occurred when the ratio of the intake of protein to carbohydrate was low; in this case, the optimum ratio was 1:10 [16].

CR did not increase lifespan in this Geometric Framework study [16]. The difference between the Geometric Framework study and previous CR studies was that mice in the Geometric Framework study were not fasting because they had ad libitum access to diluted food, while in traditional CR studies, mice are fasting in the periods between when their aliquots of food are provided. These studies support a role of fasting in the effects of standard models of CR on lifespan. They also suggest that reduction in calorie intake by itself may not be as important as previously concluded, although the effects of caloric density and indigestible fiber cannot be distinguished as they are confounded.

The mouse Geometric Framework study has reported the effects of macronutrients and energy on some of the Hallmarks of Aging (Fig. 3). A transcriptomic study of the liver showed that dietary protein was driving the expression of mitochondrial genes [161]. A subsequent study of the liver proteome showed that dietary protein was driving the abundance of mitochondrial proteins. This was associated with increased oxidative injury measured by a malondialdehyde assay [162]. In this study, low energy intake was associated with increased abundance of cellular proteins associated with RNA metabolism and the spliceosome, linked to the hallmark of proteostasis. In terms of nutrient sensing pathways, insulin levels decreased as energy intake decreased, while MTOR activity was increased in the high protein, low carbohydrate diets. FGF21, which is another nutrient sensing pathway, was only elevated at low intakes of protein, especially when coupled with elevated carbohydrate intake [134]. The population of the different types of splanchnic lymphocytes were variously influenced by dietary protein, fat, energy, and protein to carbohydrate ratio. The length of telomeres in the liver was longest in diets with low protein, high carbohydrate ratio [163]. The diversity of responses of the Hallmarks of Aging to dietary energy and macronutrients indicates that the response of lifespan to one particular dietary component is the outcome of multiple biological processes, all with differing responses to diet.

## Conclusion

Regardless of underlying mechanisms, lifespan and late-life health are strongly influenced by diet. This conclusion is supported by numerous studies in different taxa and with different nutritional interventions. Of these interventions, CR has been the focus of the most research. The fact that CR impacts all the Hallmarks of Aging can be accepted as evidence that CR acts on the aging process. Beyond calorie intake, studies based on nutritional geometry have shown that protein intake and the interaction between dietary protein and carbohydrates influence age-related health and lifespan. Obesity can be considered to be accelerated aging, and CR may improve health and increase lifespan in aging and obesity via similar mechanisms. Systematically manipulating nutrient mixtures and titrating responses across a range of diets has proved to be a useful approach for resolving some of the complexities related to diet and aging, such as comparing the roles of macronutrients and dietary energy in lifespan.
